# ﻿A genetic assessment of the population structure and demographic history of *Odontamblyopuslacepedii* (Perciformes, Amblyopinae) from the northwestern Pacific

**DOI:** 10.3897/zookeys.1088.70860

**Published:** 2022-02-28

**Authors:** Linlin Zhao, Shouqiang Wang, Fangyuan Qu, Zisha Liu, Tianxiang Gao

**Affiliations:** 1 First Institute of Oceanography, Ministry of Natural Resources, Qingdao, Shandong, 266100, China Ministry of Natural Resources Qingdao China; 2 Fisheries College, Ocean University of China, Qingdao, Shandong, 266003, China Ocean University of China Qingdao China; 3 Fishery College, Zhejiang Ocean University, Zhoushan, Zhejiang, 316004, China Zhejiang Ocean University Zhoushan China

**Keywords:** Conservation, control region, fishery management, genetic diversity, genetic structure, *Odontamblyopuslacepedii*, population demography

## Abstract

Coupled with geological and geographical history, climatic oscillations during the Pleistocene period had remarkable effects on species biodiversity and distribution along the northwestern Pacific. To detect the population structure and demographic history of *Odontamblyopuslacepedii*, 547-bp fragments of the mitochondrial DNA control region were sequenced. A low level of nucleotide diversity (0.0065 ± 0.0037) and a high level of haplotype diversity (0.98 ± 0.01) was observed. The Maximum Likelihood (ML) and Bayesian Inference phylogenetic trees showed no significant genealogical structure corresponding to sampling locations. The results of AMOVA and pairwise *F*_ST_ values revealed some significant genetic differentiation among populations, and the isolation by distance (IBD) analysis supported that the genetic differentiation was associated with the geographic distances. The demographic history of *O.lacepedii* examined by neutrality tests, mismatch distribution analysis, and Bayesian Skyline Plots (BSP) analysis suggested a sudden population expansion, and the expansion time was estimated to be around the Pleistocene. We hypothesize that the climate changes during the Pleistocene, ocean currents, and larval dispersal capabilities have played an important role in shaping contemporary phylogeographic pattern and population structure of *O.lacepedii*.

## ﻿Introduction

*Odontamblyopuslacepedii* (Temminck & Schlegel, 1845), commonly referred to as “eel goby” or “worm goby,” is an elongated, mud-dwelling benthic fish (Murdy and Shibukawa 2003). This air-breathing goby can spawn thousands of eggs at once (Dotsu and Takita 1967; Wu and Zhong 2008). However, it was misidentified in most of the Chinese literature as *O.rubicundus* (Hamilton, 1822) (Chen and Zhang 2016), which is so far distributed only in the coastal waters of India. Actually, the coastal waters and intertidal zones of East Asia are mainly inhabited by *O.lacepedii* (Gonzales et al. 2006; Chen and Zhang 2016). Previous studies of this goby have been dedicated to design models about the intertidal burrows (Gonzales et al. 2008a, b), taxonomic studies (Murdy and Shibukawa 2001; Tang et al. 2010) and phylogenetic analysis (Agorreta et al. 2013; Liu et al. 2018). However, little has been known about its population genetic structure and demographic history.

The complex interactions of post geological-history events, life history, and oceanographic condition as evolutionary processes played an important role in shaping population genetic structure and biodiversity of marine fishes (Santos et al. 2006; Hu et al. 2011; Gao et al. 2020). The East China Sea, including Yellow and Bohai seas, and South China Sea constitute the marginal oceanic regions of China. The East China Sea has one of the widest shelves in the world, and it was separated from the Pacific Ocean by the Ryukyu Islands Arc during the last glacial maximum when the sea level was 130–150 m lower than today (Xie et al. 1995; Xu and Oda 1999). However, during the postglacial warming period, the barrier disappeared (Ujiié and Ujiié 1999) with the sea level rise (Siddall et al. 2003; Liu et al. 2006), and the isolated marginal seas were reconnected (Liu et al. 2006). Those changes during glacial cycles had dramatic effects on intraspecific genetic diversity and population structure of marine species (Avise 1992; Hewitt 2000; Liu et al. 2007).

Studies have indicated that intraspecific genetic differentiation within widely distributed marine organisms is particularly reduced, mainly due to the high potential of dispersal ability over large areas (Nielsen et al. 2010; O’Donnell et al. 2017). Dispersal is very important to population biology, behavioral ecology, and conservation (Koenig et al. 1996). Most marine organisms have a pelagic larval stage that has tremendous potential for dispersal (Mora and Sale 2002). High dispersal potential may allow eggs, larvae, or adults to travel long distances, yielding high connectivity and population heterogeneity (Liu et al. 2007; O’Donnell et al. 2017). For example, larvae dispersal of *Synechogobiusommaturus* (Richardson, 1862) was inferred to promote gene flow among populations, thus having a major effect on its phylogeographic pattern (Song et al. 2010). This is not the case for *Odontamblyopuslacepedii*. The demersal eggs and benthic adults of *O.lacepedii* indicates limited swimming ability, but until now no studies about its larval dispersal ability have been reported. The otolith microchemistry analysis for this species showed that it can adapt to a wide range of salinity habitats, and the life history stages of individuals hatching in different habitats emerged as different life history types (Lu et al. 2015). Therefore, it is difficult to predict the phylogeographic pattern of *O.lacepedii*.

Although *Odontamblyopuslacepedii* has low economic value and is usually not the main fishing object, it still may experience high fishing pressure in the form of by-catch. In this study, the control region of mitochondrial DNA was employed to investigate the demographic history and the population genetic structure of *O.lacepedii* from four adjacent marginal seas, Bohai Sea, Yellow Sea, East China Sea, South China Sea, and Ariake Bay. The results of the present study will have important implications for fishery management and conservation efforts.

## ﻿Materials and methods

### ﻿Sampling and sequencing

**Figure 1.**
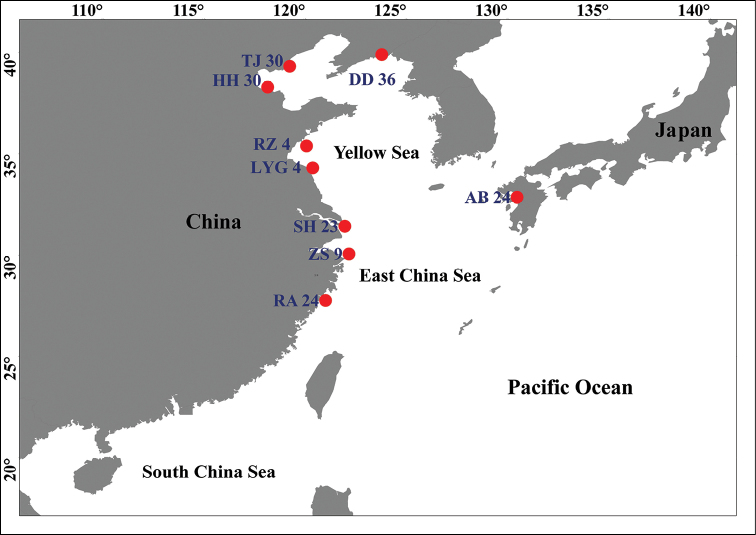
Sampling locations of *Odontamblyopuslacepedii* in the present study.

All specimens were collected along the coast of China Sea and Ariake Bay from 2013 to 2015 (Table 1; Fig. 1). Muscle tissues were preserved in 95% ethanol or directly used to extract DNA. Genomic DNA was isolated and extracted by proteinase K digestion followed by a standard phenol-chloroform method (Sambrook and Russel 2001).

**Table 1.** Sampling information of *Odontamblyopuslacepedii* examined in this study.

ID	Sampling site	Location	Sample size	Date of collection
DD	Dandong	Bohai Sea	38	2015.6–2015.12
TJ	Tianjin	Bohai Sea	30	2015.11
HH	Huanghua	Bohai Sea	30	2015.09
DY	Dongying	Bohai Sea	1	2010.05
RS	Rushan	Yellow Sea	1	2015.04
RZ	Rizhao	Yellow Sea	4	2015.03
LYG	Lianyugang	Yellow Sea	4	2014.10
SH	Shanghai	East China Sea	23	2014.11
ZS	Zhoushan	East China Sea	9	2015.12
RA	Ruian	East China Sea	24	2013.08
HZ	Huizhou	South China Sea	1	2015.04
AB	Ariake Bay	Ariake Sea	24	2014.02

A 547 bp fragment of mitochondrial DNA control region was amplified using the primers OLF: CGCTGCTTCAAAGAAGGGAGATT (forward) and OLR: CTCCCTTGTCAACTTGCCTTAG (reverse) (Liu et al. 2018). The polymerase chain reaction (PCR) was carried out in 25 μL reaction mixture containing 17.5 μL of ultrapure water, 2.5 μL of 10×PCR buffer, 2 μL of dNTPs, 1 μL of each primer (5 μM), 0.15 μL of *Taq* polymerase, and 1 μL of DNA template. The PCR amplification was carried out in a Biometra thermal cycler under conditions referred to Zhao et al (2020). PCR product was purified with a Gel Extraction Mini Kit. The purified products were used as the template DNA for cycle sequencing reactions performed using BigDye Terminator Cycle Sequencing Kit, and bi-direction sequencing was conducted on an ABI Prism 3730 automatic sequencer (Applied Biosystems) with the same primers used for sequencing as those for PCR amplification.

### ﻿Data analysis

Sequences were edited and aligned using DNASTAR software (DNASTAR, Inc., Madison, USA) and refined manually. Molecular diversity indices, such as the number of haplotypes, polymorphic sites (*S*), nucleotide diversity (*π*; Nei 1987) and haplotype diversity (*h*; Nei 1987), were calculated using Arlequin version 3.5 (Excoffier and Lischer 2010). Gene flow (*Nm*) among populations was estimated by Migrate-n version 3.6.11 (Beerli and Palcaewski 2010). Genetic differentiation between pairs of population samples was evaluated with the pairwise fixation index *F*_ST_ (Excoffier et al. 1992). The significance of the *F*_ST_ was tested by 10,000 permutations for each pairwise comparison in Arlequin version 3.5 (Excoffier and Lischer 2010). Population subdivision and significant population structure was examined using a hierarchical analysis of molecular variance (AMOVA; Excoffier et al. 1992) approximated by the Tamura and Nei model using a one-factor AMOVA with 10,000 data permutations. The populations were defined as different groups in three scenarios based on spatial distribution (Table 4). To test for isolation by distance, pairwise values of Log *F*_ST_ were plotted against geographical distance between sample sites.

The haplotype sequences were compared in MEGA11 (Tamura et al. 2021), and then the Maximum Likelihood (ML) phylogenetic tree was constructed with 1000 bootstrap replications based on distances calculated using the best selected model K2P. The phylogenetic trees were constructed with *Odontamblyopusrebecca* as the outgroup. The network of haplotypes was constructed using PopART software base on the minimum spanning network (Leigh and Bryant 2015). Demographic history was investigated using mismatch distribution and neutrality test. First is the test of selective neutrality which is performed using *D* test of Tajima (1989) and *Fs* test of Fu (1997) based on the infinite site model. Fu’s *Fs* has been shown to be especially sensitive to departure from population equilibrium as in case of a population expansion. The method to test demographic expansion is mismatch distribution which is the distribution of the observed number of differences between pairs of haplotypes based on three parameters: τ, θ_0_, and θ_1_ (τ time since expansion expressed in units of mutational time; θ before and after the population growth) (Rogers and Harpending 1992). The value of τ was transformed to estimates of real time since expansion with the equation τ = 2 μt, where τ is the crest of mismatch distribution, t is the time measured in generation since experiencing expansion, μ is the mutation rate per generation for the entire sequence. Both mismatch analysis and neutrality tests were performed in Arlequin version 3.5 (Excoffier and Lischer 2010). The population expansion time was estimated using the mutation rate of 5–10%/Myr (Song et al. 2010). BEAST v.1.7 (Drummond et al. 2012) was used to estimate the Bayesian Skyline Plots (BSP). To obtain the effective convergence, HKY + I + G model, stepwise skyline model and a strict molecular clock with 1×10^8^ iterations for Markov chain Monte Carlo (MCMC) were performed in this study. Tracer 1.7.5 software was used to generate the skyline plot (Rambaut et al. 2018).

## ﻿Results

### ﻿Genetic diversity

All sequences were aligned, and 547-bp segment of the control region was obtained for 189 specimens. A total of 83 polymorphic sites were detected and 127 haplotypes were defined (Table 2). All haplotype sequences were submitted to GenBank (Accession numbers: KX894323–KX894449). Most haplotypes were unique, of which 108 were singletons (haplotypes represented by a single sequence in the sample). Of the remaining 19 haplotypes, 13 were shared among populations, but six haplotypes belonged to one population. The most common haplotype was present in six locations with 22 individuals (7 from DD, 3 from HH, 3 from TJ, 5 from AS, 3 from SH and 1 from RS), accounting for 17.3% of all samples.

**Table 2.** Molecular diversity of *Odontamblyopuslacepedii* for seven populations, based on sequence data of the mitochondrial control region. Number of individuals (*N*), number of haplotype (*N*_h_), number of polymorphic sites (*S*), mean number of pairwise differences (*k*), haplotype diversity (*h*), nucleotide diversity (π).

Population	*N*	*N*_h_	*S*	*k*	*h*	π
DD	38	28	32	2.61±1.43	0.96±0.02	0.0048±0.0029
TJ	30	26	41	5.17±2.58	0.99±0.01	0.0095±0.0052
HH	30	28	32	4.31±2.20	0.99±0.01	0.0079±0.0045
SH	23	18	24	3.22±1.72	0.98±0.02	0.0059±0.0035
ZS	9	6	9	2.00±1.24	0.83±0.13	0.0037±0.0026
RA	24	18	24	3.41±1.81	0.92±0.05	0.0062±0.0037
AB	24	17	22	2.52±1.41	0.95±0.03	0.0046±0.0029
Total	189	127	83	3.59±1.83	0.98±0.01	0.0065±0.0037

**Figure 2.**
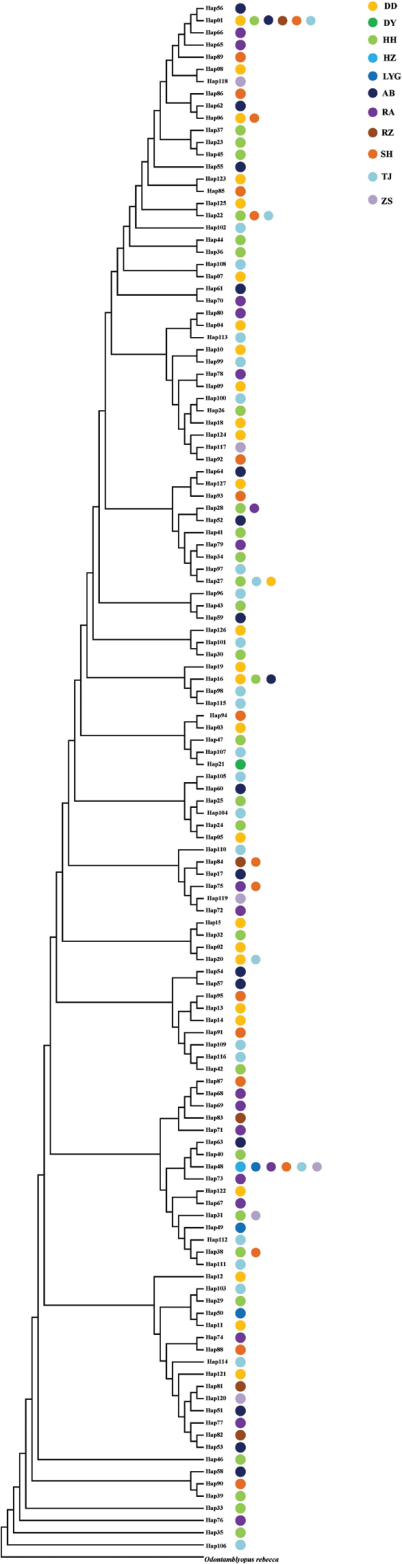
Maximum Likelihood tree is shown based on the control region haplotypes of *Odontamblyopuslacepedii.* The species of *O.rebecca* was used as the outgroup.

**Figure 3.**
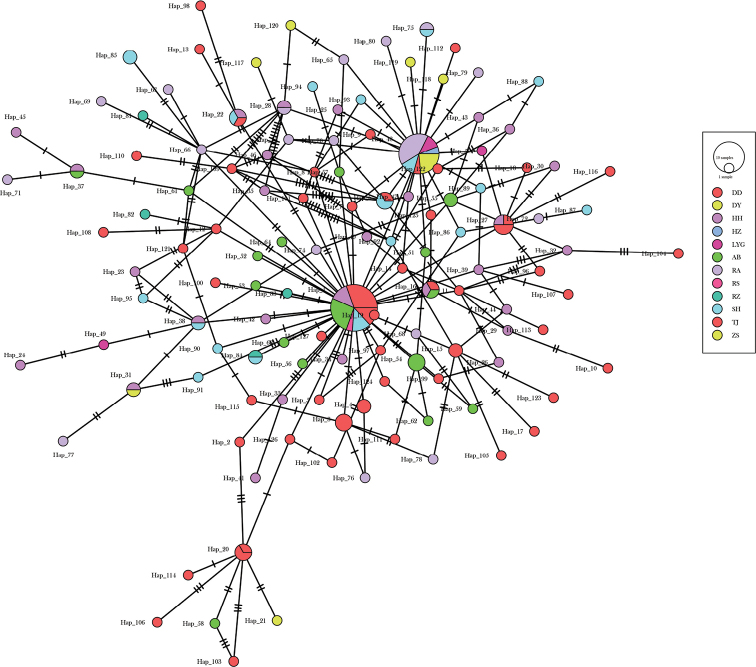
Phylogenetic network of all haplotypes.

Genetic diversity parameters for seven populations are shown in Table 2. Taxon sampling for populations DY, RS, RZ, LYG and HZ were too small and they were used only in the following phylogenetic and dynamic history analyses. A low level of nucleotide diversity and a high level of haplotype diversity was observed. Nucleotide diversity (*π*) varied from 0.0037 to 0.0095, while the haplotype diversity (*h*) ranged from 0.83 to 0.99. The phylogenetic topology based on ML analyses revealed no significant genealogical branches or clusters corresponding to sampling localities (Fig. 2). The phylogenetic network showed that haplotypes in each geographical population presented a mixed distribution pattern, and the evolutionary relationship showed multiple stellate radiation (Fig. 3).

### ﻿Population genetic structure

The pairwise *F*_ST_ values among different populations ranged from -0.009 to 0.243 (Table 3). The strong and significant genetic differentiation mainly existed among populations from different groups. The AMOVA performed under three patterns of gene pools (Table 4), and the results showed that the main variation was within populations. To test the relationship between genetic differentiation and geographic distance, IBD analysis was performed. The results showed that there was significant relationship (r = 0.54 *P* < 0.05) between Log *F*_ST_ and geographic distance, indicating that geographic distance can explain 54% of the genetic variation.

**Table 3.** The pairwise *F*_ST_ among seven populations of *Odontamblyopuslacepedii*, based on partial mitochondrial control region sequence data. Asterisks represent significance levels: *P ≤ 0.01, **P ≤ 0.001.

Population	DD	TJ	HH	SH	ZS	RA	AB
**DD**							
**TJ**	0.012						
**HH**	0.018	0.010					
**SH**	0.044**	0.032	0.015				
**ZS**	0.207**	0.103	0.097 *	0.037			
**RA**	0.103**	0.062**	0.044*	-0.001	-0.009		
**AB**	0.028**	0.030	0.033*	0.075**	0.243**	0.123**	

**Table 4.** AMOVA analysis of *Odontamblyopuslacepedii* populations based on partial mitochondrial control region sequence data.

Source of variation	d.f.	Sum of squares	Variance components	Percentage of variation	Φ-Statistics	*P*
One gene pool (DD, TJ, HH, SH, ZS, RA, AB)						
Among populations	6	23.653	0.088 Va	4.76	Φ_ST_ = 0.048	0.000
Within populations	171	299.679	1.753 Vb	95.24		
Two gene pools (DD, TJ, HH, RA, SH, ZS) (AB)						
Among groups	1	4.186	0.00904 Va	0.50	Φ_CT_ = 0.004	0.429
Among populations within groups	5	19.225	0.08448 Vb	4.64	Φ_SC_ = 0.047	0.000
Within populations	171	295.651	1.72895 Vc	94.87	Φ_ST_ = 0.051	0.000
Three gene pools (DD, TJ, HH) (SH, RA, ZS) (AB)						
Among groups	2	14.379	0.09552 Va	5.18	Φ_CT_ = 0.052	0.016
Among populations within groups	4	9.032	0.02122 Vb	1.15	Φ_SC_ = 0.012	0.045
Within populations	171	295.651	1.72895 Vc	93.67	Φ_ST_ = 0.063	0.000

### ﻿Historical demography

**Figure 4.**
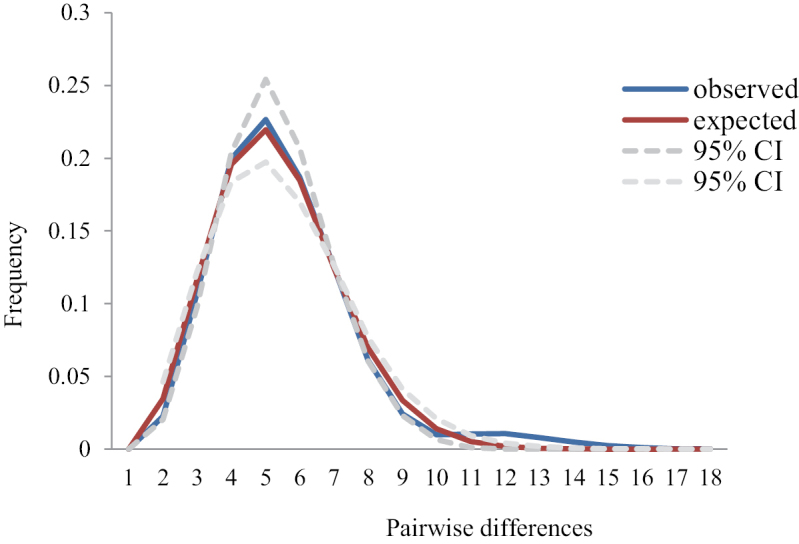
Mismatch distribution for demographic expansion based on mtDNA partial control region sequences of *Odontamblyopuslacepedii*.

**Figure 5.**
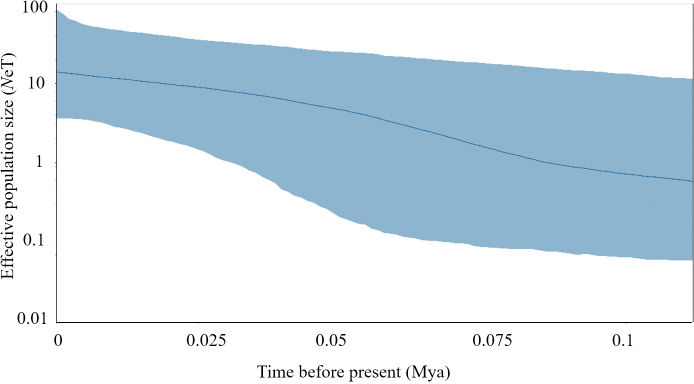
Bayesian Skyline Plots based on the mtDNA partial control region sequences of *Odontamblyopuslacepedii*.

The observed mismatch distribution of *Odontamblyopuslacepedii* for all samples is presented in Fig. 4. There are no deviations from the expected distributions (*H*ri = 0.027±0.000, *P* > 0.05), and SSD (P_SSD_ = 0.001, *P* > 0.05), and the evident unimodal mismatch distribution indicated a sudden expansion event. The Tajima’s *D* and Fu’s *Fs* tests were negative with significant *P* values (P < 0.001), which supported the hypothesis of population expansion (Table 4). The estimated time that *O.lacepedii* underwent population expansion was 61,700–123,000 years ago, based on the divergence rate of 5–10%/Myr. Estimated effective female population size after expansion (*θ*_1_) was 1.4 × 10^7^ times higher than before expansion (*θ*_0_) for *O.lacepedii*. Bayesian Skyline Plots for all samples showed late Pleistocene demographic expansion (about 100,000 years ago) (Fig. 5), which was consistent with the estimate by mismatch distribution analysis.

## ﻿Discussion

Mitochondrial DNA has been proven to be effective for population genetic analysis of marine fishes (Neethling et al. 2008; Bae et al. 2020; Zhao et al. 2020). The high level of haplotype diversity and low nucleotide diversity of *Odontamblyopuslacepedii* may be a signature of population expansion after founder events or bottlenecks (Grant and Bowen 1998; Zhang et al. 2006). The neutral test and BSP analysis supported that *O.lacepedii* may have undergone a sudden demographic expansion from its historic refugium. The time of expansion was estimated to be in the late Pleistocene. Pleistocene environmental fluctuations such as sea levels and temperatures had direct effects on species numbers, distributions, and demographics changes (Avise 2000; Gopal et al. 2006; Heyden et al. 2007; Shen et al. 2011). During the last Pleistocene, lower sea levels were associated with Pleistocene glaciations, which resulted in that most of the Chinese continental shelf was exposed, and the Asian continent was separated from the Pacific by a series of marginal seas (Tamaki and Honza 1991; Xu and Oda 1999). When the glaciers retreated, with temperature and sea level rising, those populations sheltering in their ice-age refugium might have undergone a postglacial expansion into new territory. This information supports the hypothesis that *O.lacepedii* had experienced historical expansion from a glacial refugium.

**Table 5.** Tajima’s *D*, Fu’ *Fs* statistic and mismatch parameter estimates for *Odontamblyopuslacepedii* populations.

Population	Number	*D*	*P*	*Fs*	*P*	τ	Thet0	Thet1
DD	38	-2.29	0.002	-26.55	0.000	2.643	0.002	99999.000
TJ	30	-1.84	0.016	-22.09	0.000	3.768	0.687	99999.000
HH	30	-1.69	0.026	-25.75	0.000	3.691	0.000	99999.000
SH	23	-1.89	0.015	-14.44	0.000	3.248	0.000	99999.000
ZS	9	-1.82	0.010	-2.18	0.043	2.395	0.009	10.332
RA	24	-1.74	0.023	-13.10	0.000	4.346	0.002	12.336
AB	24	-2.04	0.008	-14.09	0.000	2.428	0.000	99999.000
Total*	189	-2.29	0.000	-25.86	0.000	3.359	0.012	99999.000

* Total sequences contain all the sequences (including individuals from population DY, RS, RZ, LYG and HZ).

The population structure in marine species has been assumed to have low genetic differentiation among widespread populations due to their high potential dispersal ability and the absence of obvious geographical barriers (Rivera et al. 2004; He et al. 2015). In the present study, the pairwise *F*_ST_ statistics were low and not significant between populations with close spatial distance, demonstrating high gene flow among populations of *Odontamblyopuslacepedii*. Mukai et al. (2009) found low genetic differentiation of a reef goby (*Bathygobiuscocosensis*) in the Japan-Ryukyu-Guam region, and the oceanic currents might contribute to the dispersal and migration of larvae of this species (Mukai et al. 2009). The pelagic larval dispersal ability is theoretically associated with the level of gene flow and genetic structure (Bay et al. 2006; He et al. 2015). The eggs of *O.lacepedii* were demersal and the movements of adults were restricted to a small area (Dotsu and Takita 1967; Gonzales et al. 2006), and therefore, it is likely that the adult and eggs possessed no ability to migrate long distances. Many sedentary organisms disperse primarily during the larval phase (Kochzius and Blohm 2005; Song et al. 2010). Besides, the significant population genetic differentiation was detected between different populations with long spatial distance. The isolation by distance (IBD) analysis of this study supported that the genetic differentiation was associated with the geographic distances. The dispersal ability of marine organisms will weaken as the distance increases, which often leads to the IBD patterns (Song et al. 2010).

Apart from historical events and life history (Li et al. 2015), environmental factors, especially marine currents, may greatly influence the genetic population structures of marine species (He et al. 2015). In this study, pelagic larval durations during from July to October were predicted to be linked by the connectivity of ocean currents (Dotsu 1957). The Kuroshio Current flows in a northerly direction, and velocities were commonly recorded as 15–40 cm/s (Guan 1978). The West Korea Coastal Current flows in a southerly direction along the west Korean Peninsula, making a confluence with the Kuroshio Current into Tsushima Strait (Wei 2004). The prevailing wind may enhance the marine current dispersal distance. The ample gene flow among populations implies that the pelagic larval may be transported by the water exchange on these powerfully oceanic currents and therefore, the connectivity among populations should be high.

## ﻿Conclusions

Historical events of the Pleistocene, ocean currents, and larval dispersal capabilities have played an important role in shaping the contemporary phylogeographic patterns and population structures of *Odontamblyopuslacepedii*. With modern exploitation and habitat destroyed, *O.lacepedii* may experience high fishing pressure. The results of the present study have important implications for fisheries management and conservation efforts and for other species with similar life history characters.
